# No genetic link between Parkinson’s disease and SARS-CoV-2 infection: a two-sample Mendelian randomization study

**DOI:** 10.3389/fneur.2024.1393888

**Published:** 2024-06-28

**Authors:** Xiaohua Hu, Yutong Li, Hua Qu, Chunying He, Zhiyan Chen, Min Zhan, Yida Du, Huan Wang, Wenjie Chen, Linjuan Sun, Xia Ning

**Affiliations:** ^1^Graduate School, China Academy of Chinese Medical Sciences, Beijing, China; ^2^Graduate School, Beijing University of Chinese Medicine, Beijing, China; ^3^National Research Center for Cardiovascular Diseases of Traditional Chinese, Beijing, China; ^4^Xiyuan Hospital, Chinese Academy of Traditional Chinese Medicine, Beijing, China; ^5^Ezhou Traditional Chinese Medicine Hospital, Ezhou, China

**Keywords:** Parkinson’s disease, SARS-CoV-2, Mendelian randomization study, infection, genetic link

## Abstract

**Objective:**

Existing literature has not clearly elucidated whether SARS-CoV-2 infection increases the incidence of Parkinson’s disease or if Parkinson’s disease patients are more susceptible to the effects of SARS-CoV-2 infection. To clarify the issue, this study employs a genetic epidemiological approach to investigate the association.

**Methods:**

This study utilizes a two-sample Mendelian randomization analysis. The primary analysis employs the inverse variance-weighted (IVW) method, supplemented by secondary analyses including MR-Egger regression, weighted median, IVW radial method, and weighted mode, to evaluate the bidirectional causal relationship between Parkinson’s disease and SARS-CoV-2 infection.

**Results:**

IVW results showed no genetic causality between SARS-CoV-2 susceptibility, hospitalization rate and severity and Parkinson’s disease. (IVW method: *p* = 0.408 OR = 1.10 95% CI: 0.87 ~ 1.39; *p* = 0.744 OR = 1.11 95% CI: 0.94 ~ 1.09; *p* = 0.436 OR = 1.05 95% CI: 0.93 ~ 1.17). Parkinson’s disease was not genetically associated with susceptibility to new crown infections, hospitalization rates, and severity (IVW method: *p* = 0.173 OR = 1.01 95% CI: 0.99 ~ 1.03; *p* = 0.109 OR = 1.05 95% CI: 0.99 ~ 1.12; *p* = 0.209 OR = 1.03 95% CI: 0.99 ~ 1.07). MR-Egger regression, weighted median, IVW radial method, and weighted mode results are consistent with the results of the IVW method.

**Conclusion:**

This study does not support a genetic link between Parkinson’s disease and SARS-CoV-2 infection, and the association observed in previous cohort studies and observational studies may be due to other confounding factors.

## Introduction

Severe acute respiratory syndrome coronavirus 2 (SARS-CoV-2) is a highly pathogenic virus responsible for COVID-19 infections. Beyond respiratory symptoms, it infiltrates the nervous system via the respiratory tract, a phenomenon increasingly supported by research (1). The hallmark loss of smell in early Parkinson’s disease is also a prevalent symptom of COVID-19. The virus’s potential neuroinvasive nature suggests it may breach the central nervous system through retrograde infection via the olfactory system, blood–brain barrier disruption, and other routes (2, 3). This process affects the structure, metabolism, and function of the brain (4), resulting in disorders of the central nervous system (e.g., headaches, strokes, dizziness, fainting, and seizures) and the peripheral nervous system (deafness, loss of smell, and neuropathic pain) (5, 6). Clinical investigations have indicated elevated levels of anti-coronavirus antibodies in the cerebrospinal fluid of Parkinson’s disease patients compared to healthy individuals, hinting at a potential association between SARS-CoV-2 infection and Parkinson’s development (7, 8). Bioinformatics studies have delved into the SARS-CoV-2 infection-Parkinson’s relationship, revealing potential links related to oxidative stress, cytokine storms, and T cell activation triggered by misfolded proteins (9).

Currently, there have been several reported cases of Parkinson’s disease occurring during or after the process of SARS-CoV-2 infection (7, 10). However, currently, there is no evidence to suggest whether SARS-CoV-2 infection can increase the incidence of Parkinson’s disease. Multiple research reports indicate that patients with Parkinson’s disease are particularly prone to experiencing exacerbated symptoms due to SARS-CoV-2 infection (11), and SARS-CoV-2 infection can increase the mortality rate of Parkinson’s disease (12, 13), especially in later stages of weakness, comorbidities, etc., which also increase the risk of hospitalization and death among Parkinson’s disease patients due to SARS-CoV-2 infection (14). Furthermore, cohort studies indicate that the rate of SARS-CoV-2 infection among hospitalized Parkinson’s patients is higher than among non-Parkinson’s disease patients (15). However, some studies also suggest that there is no direct correlation between COVID-19 and Parkinson’s disease (15, 16), with the infection rate of COVID-19 in Parkinson’s patients showing no significant difference from that in non-Parkinson’s patients (17). The causal inferences drawn from these observational studies are constrained by limited reliability, primarily due to unidentified confounding factors. Consequently, the definitive causal relationship between SARS-CoV-2 infection and Parkinson’s disease remains elusive. It is imperative to elucidate the connection between SARS-CoV-2 infection and Parkinson’s to advance research on the diagnosis, treatment, rehabilitation, and care of Parkinson’s patients afflicted with COVID-19 pneumonia. Hence, this study employed Mendelian randomization to explore the bidirectional causal relationship between SARS-CoV-2 infection and Parkinson’s disease. It separately examined the impacts of SARS-CoV-2 infection on Parkinson’s and vice versa.

Unlike conventional epidemiological studies, Mendelian randomization leverages pooled data from genome-wide association studies (GWAS), utilizing genetic variables to ascertain the existence of a causal relationship between exposure and outcome data (18). In Mendelian randomization studies, the selection of genetic instrumental variables is crucial. Effective genetic instrumental variables must meet the following three assumptions: (1) they must be closely related to the exposure, (2) the instrumental variables must not be related to confounding factors, and (3) the instrumental variables must be related to the outcome only through the exposure (19). These three assumptions ensure that the selected instrumental variables influence the outcome solely through the exposure, thereby avoiding the impact of variables associated with both the exposure and the outcome. This method can exclude potential confounding factors, enhancing the robustness against reverse causality (20). Moreover, Mendelian randomization leverages genetic variation to simulate natural randomization experimental conditions, enabling more statistically meaningful causal inferences. Thus, utilizing Mendelian randomization to assess the association between SARS-CoV-2 infection and Parkinson’s may offer a more precise and potent investigative approach.

### SNP screening

All analyses in this study relied on publicly available data and, therefore, did not necessitate a separate ethical review. The study utilized summary statistics from genome-wide association studies to conduct Mendelian randomization in a European population. Specifically, the latest R7 version of the genetic tool from the COVID-19 Pneumonia Host Genetics Initiative was selected, representing the most significant GWAS data for COVID-19 pneumonia. Three sets of genetic variables were employed for analysis: SARS-CoV-2 infection, hospitalized COVID-19 cases, and severe respiratory-confirmed COVID-19 cases. Hospitalized COVID-19 cases referred to patients admitted with coronavirus-associated symptoms and laboratory-confirmed SARS-CoV-2 infection, while severe respiratory-confirmed COVID-19 cases involved patients requiring respiratory support or succumbing to laboratory-confirmed SARS-CoV-2 infection. For the exposure factor, Parkinson’s disease, a case–control meta-analysis encompassing 449,056 patients published by the International Parkinson’s Disease Consortium in 2019 was utilized. Specific data sources are delineated in Table 1.

**Table 1:** Detailed information on used studies.

Phenotype	Sexes	Author, published year	Sample size	Cases	Controls	ID	Source
European population							
SARS-CoV-2 infection	Combined	NA	2,297,856	122,616	2,475,240	NA	https://www.covid19hg.org/results/r7/
Hospitalized COVID-19	Combined	NA	2,095,324	32,519	2,062,805	NA	
Severe COVID-19	Combined	NA	1,086,211	13,769	1,072,442	NA	
Parkinson’s disease	Combined	Nalls et al. (2019)	482,730	33,674	449,056	ieu-b-7	https://gwas.mrcieu.ac.uk/datasets/ieu-b-7/

In this study, the instrumental variable was the SNP associated with exposure significantly linked to the outcome (*p* < 5 × 10–8), with an *r*^2^ < 0.001, and a distance of 10,000 kb to mitigate the effects of chain imbalance on analysis results. The F-statistic of each instrumental variable was computed to assess its statistical strength and prevent weak instrumental variable bias (21). A F-statistic below 10 indicates weak instrumental bias. The F-statistic data of the genetic tools selected in this study are presented in Supplementary Table 1, with F-statistics ranging between 29.788 and 844.448, indicating no weak instrumental bias. Furthermore, confounders associated with exposure and outcome in prior META analysis studies were compiled in this study, and SNPs associated with these confounders were removed via the PhenoScanner website to mitigate potential pleiotropy, as detailed in Supplementary Table 2.

### Statistical analysis

Statistical analyses were performed using R (version 4.2.1) software with the TwoSampleMR (version 0.5.6) and MRPRESSO (version 1.0) packages.

In this two-sample Mendelian randomization (MR) analysis, the primary statistical method employed was the inverse variance weighting method (*p* < 0.05 indicating statistical significance). Additionally, MR-Egger regression, weighted median, IVW radial method, and weighted mode were utilized as supplementary analysis techniques. To assess heterogeneity, Cochran’s Q test was utilized. In cases where heterogeneity was observed (*p* < 0.05), the random effects model was applied for statistical analysis. Conversely, if no heterogeneity was detected, the fixed effects model was employed for analysis. For the assessment of horizontal pleiotropy, the MR-Egger intercept test was employed (13, 22), with *p* < 0.05 indicating the presence of horizontal pleiotropy. Furthermore, MR-PRESSO was utilized to identify and eliminate horizontal multivariate outliers. Finally, sensitivity analysis was conducted using the leave-one-out method to ensure that individual single nucleotide polymorphisms (SNPs) did not unduly influence the results.

## Results

Through rigorous exclusion criteria, instrument variable information has been added to Appendix 1 of this study.

**Figure 1:**
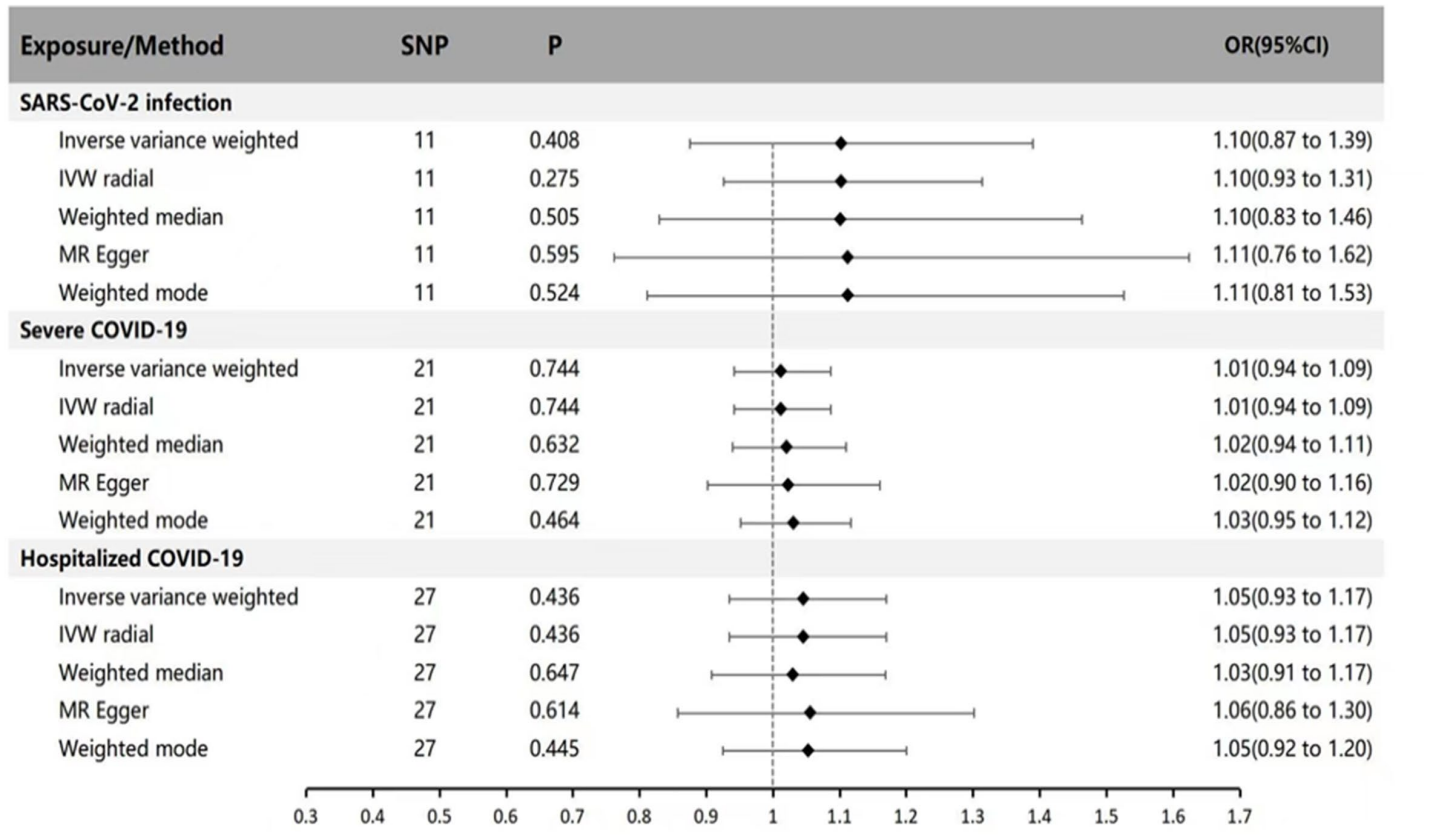
MR estimates of genetically predicted susceptibility, hospitalization, and severity of COVID-19 on the risk of Parkinson’s disease. The inverse variance weighted method is considered the main method. MR, Mendelian randomization; SNP, Single nucleotide polymorphism; P, *p*-value; OR, odds ratio; CI, Confidence interval.

In the forward MR analysis (with SARS-CoV-2 infection, Hospitalized COVID-19, and Severe COVID-19 as exposures, and Parkinson’s disease as the outcome), we separately analyzed the genetic causal relationship between COVID-19 infection, severe respiratory confirmed COVID-19, hospitalized COVID-19, and Parkinson’s disease. IVW results indicate that SARS-CoV-2 infection, Hospitalized COVID-19, and Severe COVID-19 cases do not affect susceptibility to Parkinson’s disease (*p* = 0.408 OR = 1.10 95% CI: 0.87 ~ 1.39; *p* = 0.744 OR = 1.11 95% CI: 0.94 ~ 1.09; *p* = 0.436 OR = 1.05 95% CI: 0.93 ~ 1.17). We also applied MR-Egger regression, weighted median, IVW radial method, and weighted mode for supplementary validation, and the results were not statistically significant (*p* > 0.05), consistent with the IVW results (see Figure 1 for specific results).

**Figure 2:**
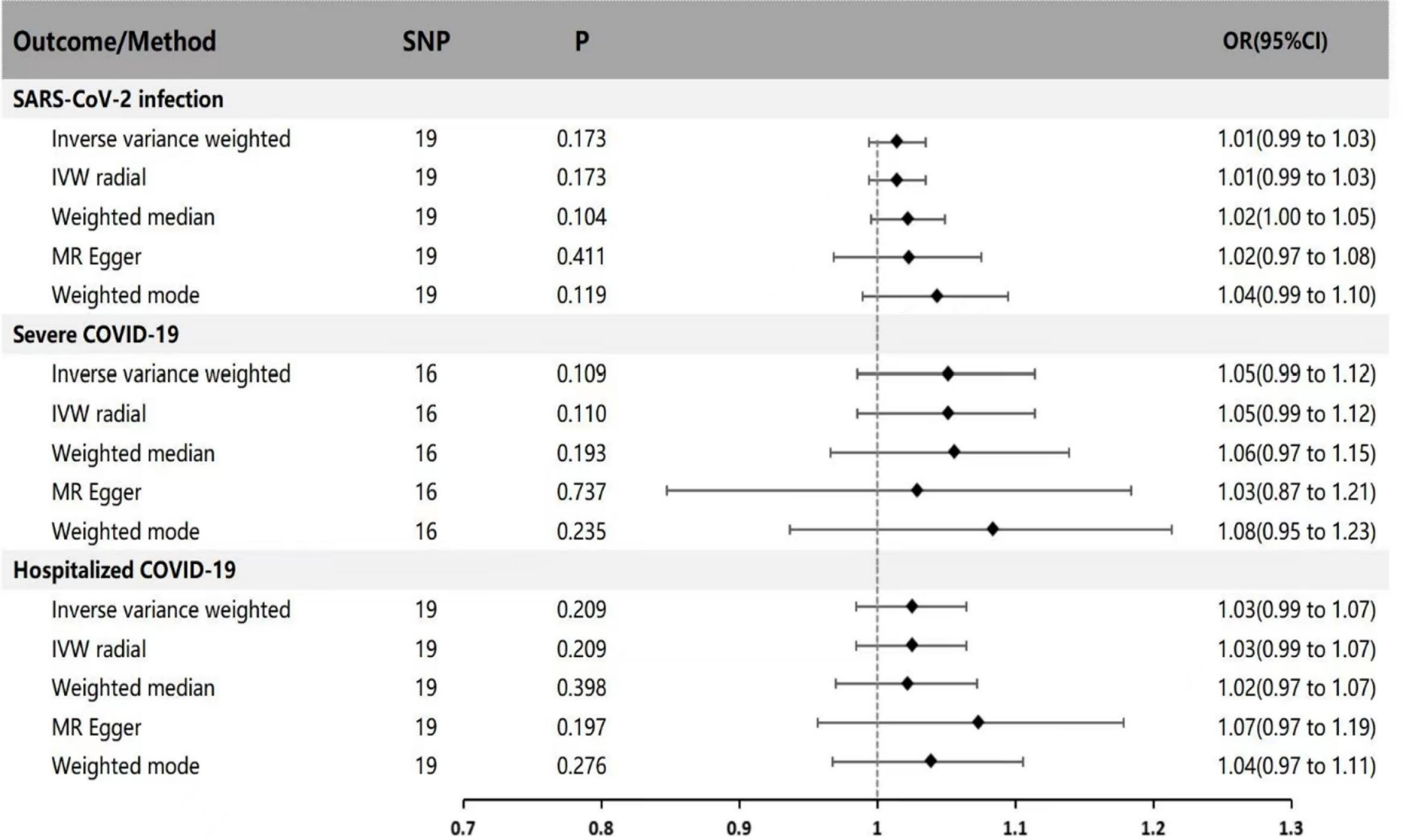
MR estimates of genetically predicted risk of Parkinson’s disease on susceptibility, hospitalization, and severity of COVID-19. The inverse variance weighted method is considered the main method. MR, Mendelian randomization; SNP, Single nucleotide polymorphism; P, *p*-value; OR, odds ratio; CI, Confidence interval.

In the reverse MR analysis (with Parkinson’s disease as exposure and SARS-CoV-2 infection, Hospitalized COVID-19, and Severe COVID-19 as outcomes), IVW results indicate no genetic association between Parkinson’s disease and SARS-CoV-2 infection, Hospitalized COVID-19, and Severe COVID-19 (IVW method: *p* = 0.173 OR = 1.01 95% CI: 0.99 ~ 1.03; *p* = 0.109 OR = 1.05 95% CI: 0.99 ~ 1.12; *p* = 0.209 OR = 1.03 95% CI: 0.99 ~ 1.07). Supplementary validation with MR-Egger regression, weighted median, IVW radial method, and weighted mode yielded results consistent with the IVW method (*p* > 0.05), indicating no genetic causal relationship (see Figure 2 for specific results).

**Figure 3:**
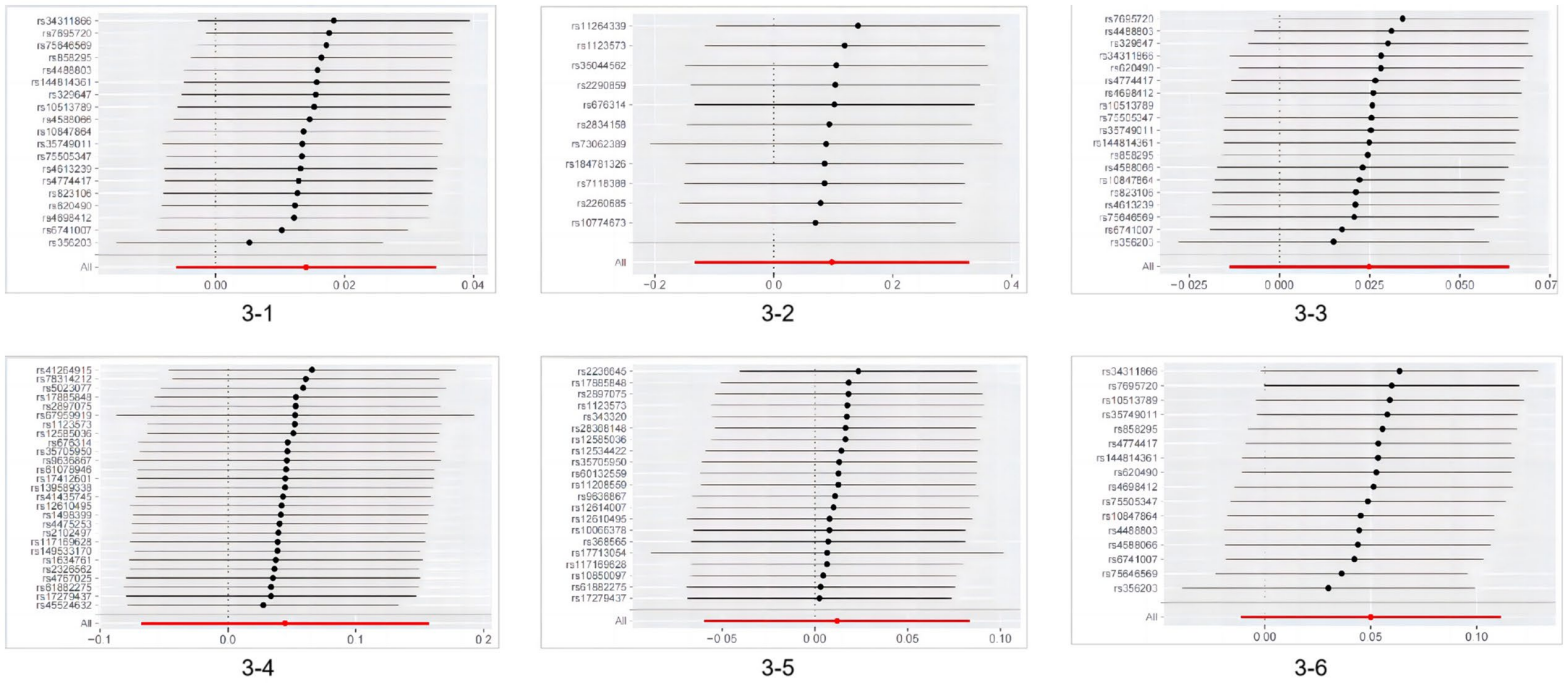
Results of MR leave-one-out sensitivity analysis. 3-1MR leave−one−out sensitivity analysis for ‘Parkinson’s disease’ on ‘SARS-CoV-2 infection’; 3–2 MR leave−one−out sensitivity analysis for ‘SARS-CoV-2 infection’ on ‘Parkinson’s disease’; 3-3MR leave−one−out sensitivity analysis for ‘Parkinson’s disease’ on ‘Hospitalized COVID-19’; 3-4MR leave−one−out sensitivity analysis for ‘Severe COVID-19’ on ‘Parkinson’s disease’; 3–5 MR leave−one−out sensitivity analysis for ‘Severe COVID-19’ on ‘Parkinson’s disease’; 3–6 MR leave−one−out sensitivity analysis for ‘Parkinson’s disease’ on ‘Severe COVID-19’ MR, Mendelian randomization.

According to Cochran’s Q test, except for the heterogeneity observed in hospitalized COVID-19 cases with Parkinson’s disease, the *p*-values for all other groups were >0.05, indicating no evidence of heterogeneity. Based on the MR-Egger intercept test results, no horizontal pleiotropy was found (*p* > 0.360), as detailed in Table 2. No heterogeneity was detected in sensitivity analysis, and individual SNPs did not independently affect the causal relationship (as shown in Figure 3).

**Table 2:** Results of Cochran’s Q statistics and MR-Egger intercept analyses.

Exposure	Outcome	Cochran’s Q (*p* value)	MR-Egger intercept (*p* value)
SARS-CoV-2 infection	Parkinson’s disease	5.738 (0.837)	−0.001 (0.955)
Severe COVID-19	Parkinson’s disease	28.064 (0.082)	−0.002 (0.840)
Hospitalized COVID-19	Parkinson’s disease	47.770 (0.006)	−0.001 (0.913)
Parkinson’s disease	SARS-CoV-2 infection	27.002 (0.079)	−0.001 (0.734)
Parkinson’s disease	Severe COVID-19	16.868 (0.327)	0.004 (0.782)
Parkinson’s disease	Hospitalized COVID-19	21.386 (0.260)	−0.008 (0.360)

## Discussion

Our findings, outlined in this paper, fail to provide substantiated evidence supporting a genetic association between SARS-CoV-2 infection and PD across various complementary MR methodologies. Similarly, the reverse MR analysis yielded no indication of a connection between genetic susceptibility to PD and SARS-CoV-2 infection.

As of now, over 20 cases of Parkinson’s disease diagnosed during or after SARS-CoV-2 infection have been reported (10, 23). Most of these patients developed the disease 4–7 weeks following SARS-CoV-2 infection. Due to the close temporal relationship between the diagnosis of new-onset Parkinson’s disease and COVID-19 pneumonia, as well as the concurrent presence of neurological disease in some cases, people have hypothesized a direct etiological link between the two. While infection has long been implicated in the pathogenesis of Parkinson’s disease (24), current evidence suggests that viral infection may predispose individuals to Parkinson’s disease rather than directly cause it (25). The angiotensin-converting enzyme receptor 2 (ACE2) serves as the primary receptor for the COVID-19 virus (3). The virus reduces ACE2 activity, exacerbating inflammatory responses and fibrosis (26). Co-expression of ACE2 with dopamine decarboxylase in the striatum and substantia nigra provides a pathophysiological basis for COVID-19 pathogen-induced Parkinson’s disease (27). Furthermore, COVID-19 virus-associated pro-inflammatory cytokines (e.g., tumor necrosis factor and interleukin 1β) may trigger a peripheral hyper-immune response, disrupting the blood–brain barrier (8). This disruption allows the infection to penetrate the central nervous system, initiating a secondary inflammatory storm (28). Neurons, with their high energy demands, are vulnerable to systemic inflammation (29). Therefore, systemic inflammation triggered by the COVID-19 virus may further contribute to neuroinflammation, causing chronic intermittent damage to human nigrostriatal dopamine neurons (1). Among the cases that have been reported, a subset of patients exhibited prodromal symptoms of Parkinson’s disease before infection with the COVID-19 virus, such as rapid eye movement, sleep behavior disorder, and constipation (10, 23). Most patients exhibited unilateral or bilateral reductions in striatal and caudate nucleus uptake. Therefore, we speculated that pathological manifestations associated with Parkinson’s disease were already present in these patients before COVID-19 infection and that the infection activated underlying neurodegenerative substrates that triggered the clinical manifestations of Parkinson’s disease. In addition, the popularity of the COVID-19 virus as a newly discovered and globally prevalent virus regarding this topic may have led to publication bias. The diagnostic criteria for Parkinson’s disease today are still based on clinical manifestations, and COVID-19 patients are diagnosed and treated by non-neurologists, who may not perform a comprehensive neurological examination at the time of diagnosis and admission to the hospital. Therefore, the diagnosis of Parkinson’s disease may also be insufficient (6).

There have been several cohort studies showing higher rates of SARS-CoV-2 infection in Parkinson’s disease patients than in non-Parkinson’s disease patients. A study of 64,434 Parkinson’s patients in Germany found a much higher rate of SARS-CoV-2 infection in hospitalized PD patients than in non-PD patients (6). Another multicenter data from Tosca also showed a higher prevalence of COVID-19 in the PD population (30). While several studies suggest that PD patients have worse prognoses and higher mortality rates after contracting COVID-19 (11, 31, 32). However, here are cohort studies and systematic evaluations suggesting that the prevalence of SARS-CoV-2 infection in Parkinson’s disease does not differ from that in non-Parkinson’s disease patients (33, 34). This is consistent with the findings of this paper. Differences in the results of different observational studies may be related to the following reasons: Most observational studies involve hospitalized COVID-19 patients, among whom PD patients may exhibit higher age, frailty, and a higher prevalence of comorbidities such as hypertension, cardiovascular and cerebrovascular diseases, chronic kidney disease, and diabetes compared to non-hospitalized PD patients. Therefore, higher COVID-19 prevalence may be related to age, gender, and comorbidities rather than PD *per se* (15). It has been suggested that advanced age, hypertension, and diabetes, which are risk factors for Parkinson’s disease, may also increase susceptibility to COVID-19 (34). The number of cases in studies on Tosca is small and underrepresented. The different epidemic prevention and control policies in each country and region may also lead to different SARS-CoV-2 infection rates in other areas, which is also one of the reasons for the different results. Certain antiparkinsonian drugs, such as amantadine, have antiviral effects and may have a protective effect against COVID-19. Poland reported five cases that tested positive for SARS-CoV-2 while taking amantadine, none showing signs of infectious disease (35). This may also contribute to the above contradiction.

This study assessed the relationship between three different types of SARS-CoV-2 infection and Parkinson’s in two directions, elaborating on the relationship between Parkinson’s and different outcomes and severity of SARS-CoV-2 infection. Although the robustness of the results was enhanced by using multiple statistical methods to corroborate each other, there are still areas for improvement in this study. Firstly, both the exposure and outcome results may include participants, and it is not easy to estimate their proportions. Secondly, although we selected the largest GWAS database to date, the small number of instrumental variables after screening suffers from under-representation and needs to be validated with more extensive GWAS data. Due to the lack of suitable GWAS data, this paper does not include a subgroup analysis of Parkinson’s disease. With the advancement of genome-wide association studies, future research should aim to improve the analysis of various subgroups of Parkinson’s disease to derive more accurate conclusions.

## Conclusion

The two-sample Mendelian randomization analysis of the present study does not support a genetic link between genetically postulated Parkinson’s disease and SARS-CoV-2 infection. The correlation demonstrated in previous case reports and cohort studies may be the result of confounding factors. To further validate our conclusions, we need to conduct more detailed analyses on the various subgroups of Parkinson’s disease. Additionally, more advanced research methods, more extensive GWAS databases, and more genetic tools are required for analysis.

## Data availability statement

The original contributions presented in the study are included in the article/Supplementary material, further inquiries can be directed to the corresponding authors.

## Ethics statement

Ethical approval was not required for the study involving humans in accordance with the local legislation and institutional requirements. Written informed consent to participate in this study was not required from the participants or the participants’ legal guardians/next of kin in accordance with the national legislation and the institutional requirements.

## Author contributions

XH: Writing – original draft, Writing – review & editing. YL: Writing – review & editing. HQ: Writing – review & editing. CH: Writing – review & editing. ZC: Writing – review & editing. MZ: Writing – review & editing. YD: Writing – review & editing. HW: Writing – review & editing. WC: Writing – review & editing. LS: Writing – review & editing. XN: Writing – review & editing.
