# Supplementary material for: No genetic link between Parkinson’s disease and SARS-CoV-2 infection: a two-sample Mendelian randomization study
Source: Front Neurol. 2024 Jun 28;15:1393888. doi: 10.3389/fneur.2024.1393888 (PMC11239547; doi:10.3389/fneur.2024.1393888)
Supplement: Supplementary file 1 [file Image_1.pdf]

TABLE I: SNPs information

exposure	outcome	SNP	effect_allele	other_allele	beta	eaf	se	p	R2	F
Parkinson disease	SARS-CoV-2 infection	rs10451230	T	A	-0.096	0.565	0.018	4.42E-08	6.23E-05	30.093
Parkinson disease	SARS-CoV-2 infection	rs10513789	G	T	-0.160	0.183	0.022	3.18E-13	1.10E-04	53.110
Parkinson disease	SARS-CoV-2 infection	rs10847864	T	G	0.127	0.363	0.018	9.81E-13	1.05E-04	50.656
Parkinson disease	SARS-CoV-2 infection	rs144814361	T	C	0.441	0.017	0.068	9.07E-11	8.72E-05	42.078
Parkinson disease	SARS-CoV-2 infection	rs329647	C	G	-0.113	0.666	0.018	1.94E-10	8.39E-05	40.515
Parkinson disease	SARS-CoV-2 infection	rs34311866	C	T	0.227	0.196	0.023	7.97E-23	2.00E-04	96.737
Parkinson disease	SARS-CoV-2 infection	rs356203	T	C	-0.240	0.617	0.018	3.01E-41	3.76E-04	181.492
Parkinson disease	SARS-CoV-2 infection	rs35749011	A	G	0.751	0.019	0.066	5.02E-30	2.69E-04	129.800
Parkinson disease	SARS-CoV-2 infection	rs4488803	A	G	-0.114	0.375	0.020	1.08E-08	6.75E-05	32.587
Parkinson disease	SARS-CoV-2 infection	rs4588066	A	G	0.105	0.326	0.018	4.45E-09	7.15E-05	34.532
Parkinson disease	SARS-CoV-2 infection	rs4613239	G	C	0.178	0.133	0.025	6.21E-13	1.07E-04	51.747
Parkinson disease	SARS-CoV-2 infection	rs4698412	A	G	0.126	0.553	0.017	7.05E-14	1.16E-04	56.071
Parkinson disease	SARS-CoV-2 infection	rs4774417	A	G	0.105	0.740	0.019	4.63E-08	6.22E-05	30.021
Parkinson disease	SARS-CoV-2 infection	rs620490	G	T	-0.117	0.276	0.019	6.46E-10	7.91E-05	38.179
Parkinson disease	SARS-CoV-2 infection	rs6741007	G	T	-0.123	0.451	0.018	2.09E-12	1.03E-04	49.642
Parkinson disease	SARS-CoV-2 infection	rs75505347	T	C	0.392	0.020	0.067	6.12E-09	7.00E-05	33.774
Parkinson disease	SARS-CoV-2 infection	rs75646569	G	T	0.192	0.112	0.027	5.62E-13	1.07E-04	51.883
Parkinson disease	SARS-CoV-2 infection	rs7695720	C	A	-0.126	0.209	0.021	1.53E-09	7.54E-05	36.405
Parkinson disease	SARS-CoV-2 infection	rs823106	C	G	-0.149	0.849	0.024	4.10E-10	8.07E-05	38.971
Parkinson disease	SARS-CoV-2 infection	rs858295	G	A	-0.104	0.395	0.018	3.83E-09	7.22E-05	34.850
Parkinson disease	Severe COVID-19	rs10451230	T	A	-0.096	0.565	0.018	4.42E-08	6.23E-05	30.093
Parkinson disease	Severe COVID-19	rs10513789	G	T	-0.160	0.183	0.022	3.18E-13	1.10E-04	53.110
Parkinson disease	Severe COVID-19	rs10847864	T	G	0.127	0.363	0.018	9.81E-13	1.05E-04	50.656
Parkinson disease	Severe COVID-19	rs144814361	T	C	0.441	0.017	0.068	9.07E-11	8.72E-05	42.078

Parkinson disease	Severe COVID-19	rs329647	C	G	-0.113	0.666	0.018	1.94E-10	8.39E-05	40.515
Parkinson disease	Severe COVID-19	rs34311866	C	T	0.227	0.196	0.023	7.97E-23	2.00E-04	96.737
Parkinson disease	Severe COVID-19	rs356203	T	C	-0.240	0.617	0.018	3.01E-41	3.76E-04	181.492
Parkinson disease	Severe COVID-19	rs35749011	A	G	0.751	0.019	0.066	5.02E-30	2.69E-04	129.800
Parkinson disease	Severe COVID-19	rs4488803	A	G	-0.114	0.375	0.020	1.08E-08	6.75E-05	32.587
Parkinson disease	Severe COVID-19	rs4588066	A	G	0.105	0.326	0.018	4.45E-09	7.15E-05	34.532
Parkinson disease	Severe COVID-19	rs4613239	G	C	0.178	0.133	0.025	6.21E-13	1.07E-04	51.747
Parkinson disease	Severe COVID-19	rs4698412	A	G	0.126	0.553	0.017	7.05E-14	1.16E-04	56.071
Parkinson disease	Severe COVID-19	rs4774417	A	G	0.105	0.740	0.019	4.63E-08	6.22E-05	30.021
Parkinson disease	Severe COVID-19	rs620490	G	T	-0.117	0.276	0.019	6.46E-10	7.91E-05	38.179
Parkinson disease	Severe COVID-19	rs6741007	G	T	-0.123	0.451	0.018	2.09E-12	1.03E-04	49.642
Parkinson disease	Severe COVID-19	rs75505347	T	C	0.392	0.020	0.067	6.12E-09	7.00E-05	33.774
Parkinson disease	Severe COVID-19	rs75646569	G	T	0.192	0.112	0.027	5.62E-13	1.07E-04	51.883
Parkinson disease	Severe COVID-19	rs7695720	C	A	-0.126	0.209	0.021	1.53E-09	7.54E-05	36.405
Parkinson disease	Severe COVID-19	rs823106	C	G	-0.149	0.849	0.024	4.10E-10	8.07E-05	38.971
Parkinson disease	Severe COVID-19	rs858295	G	A	-0.104	0.395	0.018	3.83E-09	7.22E-05	34.850
Parkinson disease	Hospitalized COVID-19	rs10451230	T	A	-0.096	0.565	0.018	4.42E-08	6.23E-05	30.093
Parkinson disease	Hospitalized COVID-19	rs10513789	G	T	-0.160	0.183	0.022	3.18E-13	1.10E-04	53.110
Parkinson disease	Hospitalized COVID-19	rs10847864	T	G	0.127	0.363	0.018	9.81E-13	1.05E-04	50.656
Parkinson disease	Hospitalized COVID-19	rs144814361	T	C	0.441	0.017	0.068	9.07E-11	8.72E-05	42.078
Parkinson disease	Hospitalized COVID-19	rs329647	C	G	-0.113	0.666	0.018	1.94E-10	8.39E-05	40.515
Parkinson disease	Hospitalized COVID-19	rs34311866	C	T	0.227	0.196	0.023	7.97E-23	2.00E-04	96.737
Parkinson disease	Hospitalized COVID-19	rs356203	T	C	-0.240	0.617	0.018	3.01E-41	3.76E-04	181.492
Parkinson disease	Hospitalized COVID-19	rs35749011	A	G	0.751	0.019	0.066	5.02E-30	2.69E-04	129.800
Parkinson disease	Hospitalized COVID-19	rs4488803	A	G	-0.114	0.375	0.020	1.08E-08	6.75E-05	32.587
Parkinson disease	Hospitalized COVID-19	rs4588066	A	G	0.105	0.326	0.018	4.45E-09	7.15E-05	34.532

Parkinson disease	Hospitalized COVID-19	rs4613239	G	C	0.178	0.133	0.025	6.21E-13	1.07E-04	51.747
Parkinson disease	Hospitalized COVID-19	rs4698412	A	G	0.126	0.553	0.017	7.05E-14	1.16E-04	56.071
Parkinson disease	Hospitalized COVID-19	rs4774417	A	G	0.105	0.740	0.019	4.63E-08	6.22E-05	30.021
Parkinson disease	Hospitalized COVID-19	rs620490	G	T	-0.117	0.276	0.019	6.46E-10	7.91E-05	38.179
Parkinson disease	Hospitalized COVID-19	rs6741007	G	T	-0.123	0.451	0.018	2.09E-12	1.03E-04	49.642
Parkinson disease	Hospitalized COVID-19	rs75505347	T	C	0.392	0.020	0.067	6.12E-09	7.00E-05	33.774
Parkinson disease	Hospitalized COVID-19	rs75646569	G	T	0.192	0.112	0.027	5.62E-13	1.07E-04	51.883
Parkinson disease	Hospitalized COVID-19	rs7695720	C	A	-0.126	0.209	0.021	1.53E-09	7.54E-05	36.405
Parkinson disease	Hospitalized COVID-19	rs823106	C	G	-0.149	0.849	0.024	4.10E-10	8.07E-05	38.971
Parkinson disease	Hospitalized COVID-19	rs858295	G	A	-0.104	0.395	0.018	3.83E-09	7.22E-05	34.850
SARS-CoV-2 infection	Parkinson disease	rs10774673	C	T	0.029	0.675	0.005	2.14E-09	3.51E-04	35.839
SARS-CoV-2 infection	Parkinson disease	rs1123573	A	G	-0.026	0.379	0.005	3.16E-08	3.10E-04	30.608
SARS-CoV-2 infection	Parkinson disease	rs11264339	C	T	-0.035	0.495	0.005	1.62E-14	5.77E-04	58.942
SARS-CoV-2 infection	Parkinson disease	rs184781326	A	G	-0.069	0.047	0.012	3.42E-09	3.66E-04	34.924
SARS-CoV-2 infection	Parkinson disease	rs2260685	T	C	0.033	0.474	0.005	1.01E-12	5.25E-04	50.822
SARS-CoV-2 infection	Parkinson disease	rs2290859	C	T	-0.051	0.351	0.005	1.32E-25	1.07E-03	109.402
SARS-CoV-2 infection	Parkinson disease	rs2834158	T	C	-0.041	0.661	0.005	8.51E-17	6.81E-04	69.287
SARS-CoV-2 infection	Parkinson disease	rs35044562	A	G	0.128	0.078	0.008	1.64E-51	2.23E-03	227.968
SARS-CoV-2 infection	Parkinson disease	rs676314	A	G	0.028	0.326	0.005	1.02E-08	3.23E-04	32.807
SARS-CoV-2 infection	Parkinson disease	rs7118388	A	G	0.027	0.506	0.005	4.71E-09	3.35E-04	34.305
SARS-CoV-2 infection	Parkinson disease	rs73062389	G	A	0.200	0.054	0.010	7.61E-92	4.33E-03	413.134
Severe COVID-19	Parkinson disease	rs10066378	T	C	0.118	0.116	0.021	1.96E-08	2.64E-03	31.529
Severe COVID-19	Parkinson disease	rs10850097	C	T	0.095	0.671	0.015	1.71E-10	3.41E-03	40.769
Severe COVID-19	Parkinson disease	rs11208559	C	G	0.103	0.291	0.017	2.17E-09	3.32E-03	35.810
Severe COVID-19	Parkinson disease	rs1123573	A	G	-0.106	0.370	0.015	2.80E-12	4.47E-03	48.816
Severe COVID-19	Parkinson disease	rs117169628	G	A	0.157	0.137	0.020	4.36E-15	5.14E-03	61.517

Severe COVID-19	Parkinson disease	rs12534422	C	T	0.086	0.303	0.015	1.34E-08	2.70E-03	32.264
Severe COVID-19	Parkinson disease	rs12585036	C	T	0.141	0.213	0.017	2.32E-16	5.62E-03	67.296
Severe COVID-19	Parkinson disease	rs12610495	A	G	0.242	0.313	0.016	3.05E-51	2.04E-02	226.709
Severe COVID-19	Parkinson disease	rs12614007	G	A	0.094	0.749	0.017	2.46E-08	2.60E-03	31.090
Severe COVID-19	Parkinson disease	rs17279437	G	A	-0.172	0.101	0.025	7.29E-12	3.93E-03	46.937
Severe COVID-19	Parkinson disease	rs17713054	G	A	0.756	0.075	0.026	1.09E-185	6.62E-02	844.448
Severe COVID-19	Parkinson disease	rs17885848	C	T	0.090	0.339	0.016	7.78E-09	3.04E-03	33.325
Severe COVID-19	Parkinson disease	rs2236645	C	T	0.179	0.085	0.025	6.46E-13	4.32E-03	51.695
Severe COVID-19	Parkinson disease	rs2569703	C	G	-0.108	0.563	0.014	1.28E-14	4.93E-03	59.390
Severe COVID-19	Parkinson disease	rs28368148	C	G	0.449	0.023	0.065	7.26E-12	4.49E-03	46.945
Severe COVID-19	Parkinson disease	rs2897075	C	T	0.088	0.376	0.014	9.32E-10	3.11E-03	37.458
Severe COVID-19	Parkinson disease	rs343320	G	A	0.154	0.070	0.028	2.06E-08	2.93E-03	31.430
Severe COVID-19	Parkinson disease	rs35705950	G	T	-0.164	0.109	0.023	6.95E-13	4.33E-03	51.546
Severe COVID-19	Parkinson disease	rs368565	C	T	0.106	0.464	0.015	1.41E-12	4.60E-03	50.159
Severe COVID-19	Parkinson disease	rs60132559	C	T	0.091	0.321	0.015	1.11E-09	3.06E-03	37.124
Severe COVID-19	Parkinson disease	rs61882275	G	A	-0.126	0.343	0.015	8.08E-18	6.20E-03	73.927
Severe COVID-19	Parkinson disease	rs9636867	A	G	0.184	0.331	0.015	3.47E-34	1.21E-02	148.599
Hospitalized COVID-19	Parkinson disease	rs1123573	A	G	-0.069	0.377	0.010	4.13E-11	1.92E-03	43.552
Hospitalized COVID-19	Parkinson disease	rs117169628	G	A	0.101	0.144	0.014	1.27E-13	2.12E-03	54.889
Hospitalized COVID-19	Parkinson disease	rs12585036	C	T	0.097	0.216	0.012	9.39E-17	2.67E-03	69.085
Hospitalized COVID-19	Parkinson disease	rs12610495	A	G	0.148	0.305	0.011	4.21E-41	7.91E-03	180.282
Hospitalized COVID-19	Parkinson disease	rs139589338	A	G	0.209	0.019	0.036	4.97E-09	1.58E-03	34.200
Hospitalized COVID-19	Parkinson disease	rs149533170	G	A	0.284	0.008	0.051	2.78E-08	1.66E-03	30.854
Hospitalized COVID-19	Parkinson disease	rs1498399	A	G	0.069	0.386	0.010	8.24E-12	1.82E-03	46.703
Hospitalized COVID-19	Parkinson disease	rs1634761	C	T	-0.068	0.494	0.009	1.02E-12	1.92E-03	50.800
Hospitalized COVID-19	Parkinson disease	rs17279437	G	A	-0.114	0.107	0.017	1.37E-11	1.74E-03	45.713

Hospitalized COVID-19	Parkinson disease	rs17412601	T	C	-0.068	0.351	0.010	3.69E-11	1.70E-03	43.770
Hospitalized COVID-19	Parkinson disease	rs17885848	C	T	0.061	0.337	0.011	1.93E-08	1.45E-03	31.566
Hospitalized COVID-19	Parkinson disease	rs2102497	T	C	0.064	0.734	0.012	4.21E-08	1.33E-03	30.046
Hospitalized COVID-19	Parkinson disease	rs2326562	C	T	0.053	0.381	0.010	4.81E-08	1.13E-03	29.788
Hospitalized COVID-19	Parkinson disease	rs2897075	C	T	0.059	0.374	0.010	2.88E-09	1.37E-03	35.257
Hospitalized COVID-19	Parkinson disease	rs35705950	G	T	-0.099	0.107	0.016	1.90E-10	1.60E-03	40.569
Hospitalized COVID-19	Parkinson disease	rs41264915	A	G	-0.143	0.097	0.015	1.43E-20	3.30E-03	86.450
Hospitalized COVID-19	Parkinson disease	rs41435745	G	C	0.213	0.029	0.035	1.45E-09	1.47E-03	36.592
Hospitalized COVID-19	Parkinson disease	rs4475253	A	G	0.058	0.324	0.010	7.07E-09	1.27E-03	33.513
Hospitalized COVID-19	Parkinson disease	rs45524632	C	A	0.223	0.019	0.035	1.07E-10	1.69E-03	41.689
Hospitalized COVID-19	Parkinson disease	rs4767025	C	T	0.075	0.676	0.010	7.13E-14	2.14E-03	56.034
Hospitalized COVID-19	Parkinson disease	rs5023077	T	C	-0.066	0.491	0.010	3.79E-12	1.86E-03	48.229
Hospitalized COVID-19	Parkinson disease	rs61078946	A	T	-0.090	0.111	0.016	3.12E-08	1.20E-03	30.628
Hospitalized COVID-19	Parkinson disease	rs61882275	G	A	-0.092	0.342	0.010	2.30E-20	3.30E-03	85.504
Hospitalized COVID-19	Parkinson disease	rs676314	A	G	0.078	0.325	0.010	8.95E-15	2.31E-03	60.113
Hospitalized COVID-19	Parkinson disease	rs67959919	G	A	0.492	0.077	0.018	2.36E-173	2.96E-02	787.832
Hospitalized COVID-19	Parkinson disease	rs78314212	C	T	0.122	0.085	0.017	1.22E-12	1.92E-03	50.454
Hospitalized COVID-19	Parkinson disease	rs9636867	A	G	0.128	0.336	0.010	2.05E-36	6.05E-03	158.803

TABLE 2:Confounding factors and deleted SNPs

	Potential confounders	Author	PMID	SNP deleted
**Potential confounders of Parkinson Disease**	exposure to pesticides	A Priyadarshi et al.	11022853	
	diabetes	Dagfinn et al.	37185794	rs35265698,rs35265698,
	anxiety or depression	Bellou et al.	25139772	rs12934900,rs58879558,
	smoking	Bellou et al.	25139772	
	constipation	Bellou et al.	28688713	
**Potential confounders of COVID-19**	diabetes	Marina et al.	34446016	rs1128175
	obesity	Marina et al.	34446016	
	COPD	Marina et al.	34446016	rs12610495
	chronic kidney disease	Fang et al.	32658868	
	cardiovascular disease	Zheng et al.	32335169	rs679574,rs368565,rs2251034
	hypertension	Zheng et al.	32335169	
	Vitamin D	Kazemi et al.	33751020	
